# Oblique light incidence method to study topological defects in nematic layers with conical boundary conditions

**DOI:** 10.1038/s41598-021-96784-9

**Published:** 2021-08-31

**Authors:** Mikhail N. Krakhalev

**Affiliations:** 1 Kirensky Institute of Physics, Federal Research Center KSC SB RAS, Krasnoyarsk, Russia 660036; 2 Institute of Engineering Physics and Radio Electronics, Siberian Federal University, Krasnoyarsk, Russia 660041

**Keywords:** Polarization microscopy, Liquid crystals

## Abstract

A polarization microscopy method to investigate the orientational structures and boojums formed in the chiral and achiral nematic layers under conical (tilted) boundary conditions has been developed. Oblique light incidence on nematic layer is used, due to which the phase difference between the ordinary and extraordinary waves depends on the director’s azimuthal angle. The phase difference gets maximal when the director azimuthal angle of achiral nematic $\varphi (x,y) = 0$ and an azimuthal angle at the center of the chiral nematic layer $\varphi _0(x,y) = 0$ independently of the total twist angle $\varphi _{TOTAL}$. It has been found that the $m=+1$ boojums with the phase $\xi = \pm 90^\circ$ and $\xi = (-90^\circ + \varphi _{TOTAL}/2)$ are formed in achiral and chiral nematics, respectively, at the director tilt angle $\theta _{d/2} \cong 40^\circ$ at the interface. In addition, the defectless structure of chiral nematic with the periodically variable azimuthal director angle on the substrates has been studied.

## Introduction

Liquid crystals (LC) are anisotropic liquids with the long-range orientational molecules order. The orientation of LC molecules is described with the director $\mathbf {n}$, being the unit vector oriented along the preferred orientation of long axes of molecules^1^. A rich variety of director configurations assigned by the boundary conditions (the director orientation on the interface, anchoring energy), by material parameters of LC (elastic constants, cholesteric helix pitch), by external electric or magnetic fields is observed in LC^2^. At that, the defects of orientational structure can form due to significant distortion of the director field. This distortion inside bulk can be induced, for instance, by the magnetic (electric) field, insertion of small particles into LC, microfluidics flows, etc. Owing to elastic distortions of the director field, an interaction between the defects and/or particles occurs. This manifests itself in the effects of self-ordering of defects^3–7^ or colloidal/magnetic microparticles^8–10^, controlling of the position of microparticles^11^. It also determines group moving of defects in the external electric field^12^. Topological defects also influence the spatial and temporal dynamics of biological objects dispersed in LC^13–15^.

In nematic and cholesteric LC the point bulk (hedgehogs)^16,17^ and surface (boojums)^18^ defects, the linear defects^19–21^, the two-dimensional defects (walls)^1,2^ or the soliton-like structures^22,23^ can form. The possibility of formation and stability of various types of defects depend on the boundary conditions assigned by the substrates. So, under degenerated tangential (planar) boundary conditions (angle between the director and normal to the surface (a tilt angle) is equal to 90°) the schlieren-texture consisting of point and linear defects, the strength of which depends on the ratio of elastic constants, is formed in LC layer^24,25^. The LC is free from any defects under homeotropic anchoring of nematic (the tilt angle is equal to 0°). However, an electric field or a combination of the electric and magnetic fields applied to the LC causes a formation of point defects^26,27^, two-dimensional defects^28–30^, or soliton-like structures in the chiral nematic^31^. At homeo-planar boundary conditions, the point or linear defects can form at the substrate with planar anchoring^32–35^. The conical boundary conditions (the tilt angle different from 0° and 90° and the azimuthal orientation of the director is degenerate) are the most favourable concerning the diversity of forming defects. For example, point, linear, and two-dimensional defects appear in the nematic under conical anchoring even without external fields^36^. The structures with planar-conical anchoring have been researched earlier for the chiral nematic. In this case the periodic structure of stripes^37^, the structures with linear defects^38,39^ or domains limited by the closed linear defect with pair of point singularities^40^ have been observed. At that, the orientational structures and the topological defects in chiral nematics at conical anchoring are insufficiently studied until present time.

The LC orientational structures are mostly studied by the polarization microscopy techniques^24^. Orthoscopic microscopy is convenient to investigate the director projection configuration on the sample plane (*xy*-plane) but it does not allow to determine the director *z*-component. Three-dimensional imaging of LC orientational structure can be obtained using fluorescence confocal polarizing microscopy or the three-photon excitation fluorescence polarizing microscopy^41–44^. These methods are based on the dependence of the fluorescence intensity and polarization on the orientation of dye molecules dissolved in the LC host or the LC molecules themselves. Low birefringence of applied LCs for these methods is a prerequisite. The oblique light incidence methods (the conoscopic observations, for example) for defectless orientational structures are suitable to determine the director *z*-component^45,46^. This approach is used to measure the director’s tilt angles at substrates where the director orientation is uniform.

In this paper we have developed the oblique light incidence method for the polarising optical microscopy (POM) to study the LC orientational structures in the cells with conical anchoring on both substrates and demonstrated its capabilities as applied to the point surface defects arising in chiral and achiral nematics under such boundary conditions.

## Results

**Figure 1:**
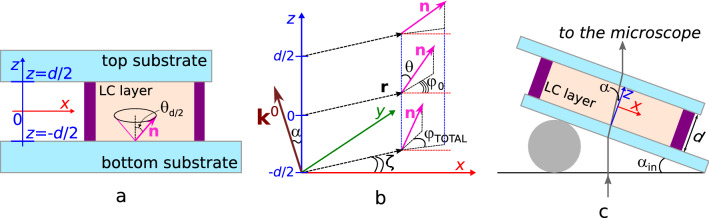
Schemes of the experimental cell (**a**), the coordinate system used to calculate phase difference $\delta$ and the director field (**b**). Position scheme of LC cell on the microscopic stage for viewing LC layer at oblique light incidence (**c**). $\theta _{d/2} = \theta _{-d/2}$ are tilt director angles at the top and bottom substrates, respectively, the vector $\mathbf {k}^0(-\sin {\alpha },0,\cos {\alpha })$ is in the plane *xOz*.

The director field within the LC cell is conveniently described by the tilt angle $\theta (x,y,z)$ and azimuthal angle $\varphi (x,y,z)$ (Fig. 1). Then, the director $\mathbf {n}$ is given by:

1 $$\begin{aligned} \left\{ \begin{array}{l} n_x = \sin {\theta (x,y,z)} \cos {\varphi (x,y,z)}\\ n_y = \sin {\theta (x,y,z)} \sin {\varphi (x,y,z)}\\ n_z = \cos {\theta (x,y,z)} \end{array} \right. . \end{aligned}$$

Generally, $\theta$ and $\varphi$ angles vary both over the LC layer thickness and within the substrate plane. It is especially shown near the defects, where the character of changing of tilt and azimuthal angles is conditioned by the defect type. Conversely, a character of angles change in the vicinity of the defect allows determining their parameters.

### Oblique light incidence method

#### The achiral nematic case

It can be assumed the tilt director angle for nematic structure far from the defects is constant and equal to the director tilt angle $\theta (x,y,z) = \theta _{d/2}$ on the substrates and $\varphi (x,y)$ azimuthal angle doesn’t depend on the coordinate *z*. Let’s consider the oblique (in the plane *xOz*) light incidence on the LC layer (Fig. 1b,c). The beam direction will be characterised by the unit vector $\mathbf {k}^0(\pm \sin {\alpha },0,\cos {\alpha })$, where “+” sign corresponds to zero azimuthal angle of vector $\mathbf {k}^0$, and “−” sign corresponds to the value of azimuthal angle of vector $\mathbf {k}^0$ equal to $\pi$. $\delta$ phase difference between ordinary and extraordinary waves in this case is equal to:

2 $$\begin{aligned} \delta = \frac{2\pi d}{\lambda \cos {\alpha }} \left( \frac{n_{\parallel } n_{\perp }}{\sqrt{n^2_{\perp }+\left( n^2_{\parallel }-n^2_{\perp }\right) \cos ^2{\theta '}}} - n_{\perp } \right) =\frac{2\pi d}{\lambda \cos {\alpha }} \Delta n , \end{aligned}$$

where $n_{\perp }$ and $n_{\parallel }$ are refractive indices for light polarized perpendicular and parallel to the director, respectively, *d* is thickness of LC cell, $\lambda$ is light wavelength, $\theta '$ is angle between the director and $\mathbf {k}^0$ vector determined by:

3 $$\begin{aligned} cos{\theta '} = \mathbf {n}\mathbf {k}^0 = \pm \sin {\alpha }\sin {\theta _{d/2}}cos{\varphi (x,y)} + \cos {\alpha }\cos {\theta _{d/2}}. \end{aligned}$$

As follows from Eq. (2) the maximal phase difference $\delta _{max}$ satisfy the condition when $\cos ^2{\theta '}$ takes the smallest value and, conversely, the minimal phase difference meets to maximal value of $\cos ^2{\theta '}$. If the condition $(\theta _{d/2} + \alpha ) \le 90^\circ$ (here we consider that $0^\circ \le \theta _{d/2} \le 90^\circ$) is satisfied, then one maximum (one minimum) of $\delta$ value satisfying to the condition $\cos {\varphi (x,y)} = +1$ ($\cos {\varphi (x,y)} = -1$) is observed only. For instance, for the case presented in Fig. 1b, the vector $\mathbf {k}^0$ has the coordinates $(-\sin {\alpha },0,\cos {\alpha })$, therefore $\delta _{max}$ corresponds to the value of azimuthal director angle $\varphi (x,y,z) = 0$, while $\delta _{min}$ is observed at $\varphi (x,y,z) = \pm 180^\circ$.

**Figure 2:**
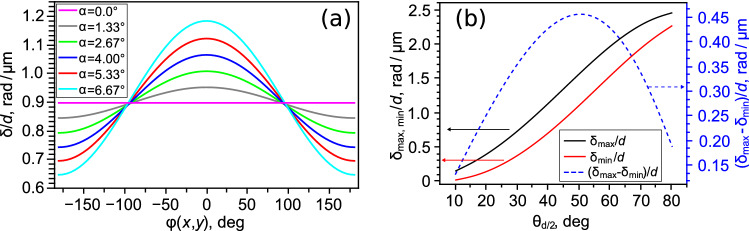
Dependencies of $\delta /d$ ratio on the angle $\varphi (x,y)$ calculated for several light incidence $\alpha$ angles and the tilt angle $\theta _{d/2} = 40^\circ$ (**a**), dependencies of $\delta _{max}/d$, $\delta _{min}/d$ and $(\delta _{max}-\delta _{max})/d$ on the tilt angle $\theta _{d/2}$ obtained for the light incidence angle $\alpha = 5.33^\circ$ (**b**). The calculations have been performed at $\lambda = 546$ nm and the refractive indices of LN-396.

Figure 2a demonstrates the dependencies of $\delta$ phase difference normalised to *d* LC layer thickness on the angle $\varphi (x,y)$. The calculations have been made for $\theta _{d/2} = 40^\circ$ and the light incidence angles appropriate to the tilt angles of LC cell $\alpha _{in} = 0^\circ , 2^\circ , 4^\circ , 6^\circ , 8^\circ$ and $10^\circ$. The angle $\alpha$ was found from the Snell’s law at the glass substrate refractive index of 1.5. As seen from the diagrams, already at the angle $\alpha \cong 2.67^\circ (\alpha _{in} = 4^\circ )$ the difference between the maximal and minimal values $(\delta _{max}-\delta _{min})/d \approx 0.21$ rad/$\upmu$m, that can be detected easily for the LC cells of a widely used thickness ($\sim 10\,\upmu$m). At that, the angles $\alpha _{in}$ specified in the calculation, on the one hand, allow free observing LC cells under a polarising optical microscope with a long-distance objective. In this case, a sharp image of a sufficiently large area can be obtained as yet. On the other hand, the small values of $\alpha$ angles allow using the method for a sufficiently wide range of $\theta _{d/2}$ angles.

As an example, Fig. 2b shows the normalised values dependencies of maximal $\delta _{max}/d$, minimal $\delta _{min}/d$ phase differences and the $(\delta _{max}-\delta _{min})/d$ on the tilt angle $\theta _{d/2}$ for $\alpha = 5.33^\circ$. It is seen that $\delta _{max, min}$ increases as $\theta _{d/2}$ rises while the difference $(\delta _{max}-\delta _{min})/d > 0.18$ rad/$\upmu$m for the tilt angles in the range of $15^\circ<\theta _{d/2}<80^\circ$ and reaches the maximum value approximately equal to 0.46 rad/$\upmu$m at angle $\theta _{d/2} \approx 50^\circ$.

#### The chiral nematic case

The dependencies of $\delta$ on $\varphi (x,y)$ for nematic have been obtained unless the azimuthal director angle depends on the *z* coordinate. Generally, the azimuthal director angle is the function of all three coordinates for nematics near the defects or for chiral nematics. Let’s consider a simple case of director $\mathbf {n}$ twisting for which the azimuthal angle $\varphi$ depends on the *z* coordinate by the linear law:

4 $$\begin{aligned} \varphi (x,y,z) = \varphi _0(x,y) + \frac{\varphi _{TOTAL}}{d}z, \end{aligned}$$

where $\varphi _0(x,y)$ is azimuthal director angle in the LC layer centre (at $z = 0$), $\varphi _{TOTAL}$ is the difference of azimuthal director angles on the top ($z = +d/2$) and bottom ($z = -d/2$) substrates (the total twist angle along the LC layer thickness *d*). If the Mauguin condition $M = 2\pi d \Delta n/(\varphi _{TOTAL} \lambda ) \gg 1$ is valid^47^, then we can assume that two linearly-polarized ordinary and extraordinary waves propagate through LC layer and $\delta$ phase difference between them for the case of the oblique light incidence at the angle $\alpha$ is given by:

5 $$\begin{aligned} \delta = \frac{2\pi n_{\perp } d}{\lambda \cos {\alpha }} \left( \int _{-\frac{1}{2}}^{\frac{1}{2}}{\frac{n_{\parallel }}{\sqrt{n^2_{\perp }+(n^2_{\parallel }-n^2_{\perp })\cos ^2{\theta '}}} \,dz'} - 1 \right) , \end{aligned}$$

where $z' = z/d$, and $\cos {\theta }'$ is found as:

6 $$\begin{aligned} cos{\theta '} = \mathbf {n}\mathbf {k}^0 = \pm \sin {\alpha }\sin {\theta _{d/2}}cos{(\varphi _0(x,y)+z'\varphi _{TOTAL})} + \cos {\alpha }\cos {\theta _{d/2}}. \end{aligned}$$

**Figure 3:**
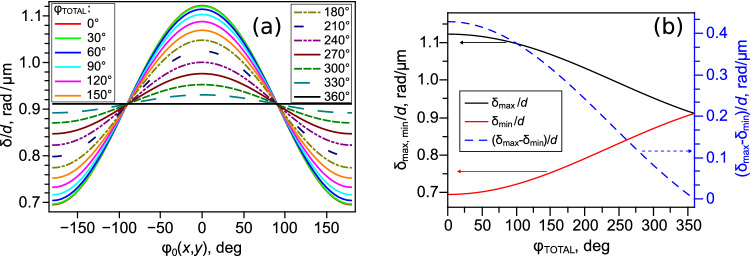
The dependencies of the ratio $\delta /d$ on the azimuthal angle $\varphi _0(x,y)$ calculated for the some $\varphi _{TOTAL}$ twist angles (**a**), the dependencies of $\delta _{max}/d$, $\delta _{min}/d$ and $(\delta _{max}-\delta _{max})/d$ on the total twist angle $\varphi _{TOTAL}$ (**b**). The calculations were performed for the angles $\alpha = 5.33^\circ$, $\theta _{d/2} = 40^\circ$, the light wavelength $\lambda = 546$ nm, and the refractive indices of LN-396.

Figure 3a presents the dependencies of $\delta /d$ on the angle $\varphi _0(x,y)$, calculated by Eq. (5) for some values of the total twist angle $\varphi _{TOTAL}$ and $\alpha = 5.33^\circ$ (as shown in Fig. 1b) at the director tilt angle $\theta _{d/2} = 40^\circ$. As can see, the presented curves have only one maximum corresponding to the azimuthal angle $\varphi _0(x,y) = 0^\circ$. As the angle $\varphi _{TOTAL}$ increases, the difference $(\delta _{max}-\delta _{min})/d$ decreases and it is equal to zero at $\varphi _{TOTAL} = 360^\circ$ (Fig. 3b). At that, the $(\delta _{max}-\delta _{min})/d$ varies from approximately 0.43 rad/$\upmu$m to 0.13 rad/$\upmu$m in the range of $\varphi _{TOTAL}$ angles from $0^\circ$ to $270^\circ$. The data shown in Fig. 3 have been calculated for $\varphi _{TOTAL}$ angles positive values (right-handed nematic). Owing to the structure symmetry relative to the layer centre, the same results are obtained for the negative angles $\varphi _{TOTAL}$ (left-handed chiral nematic).

**Figure 4:**
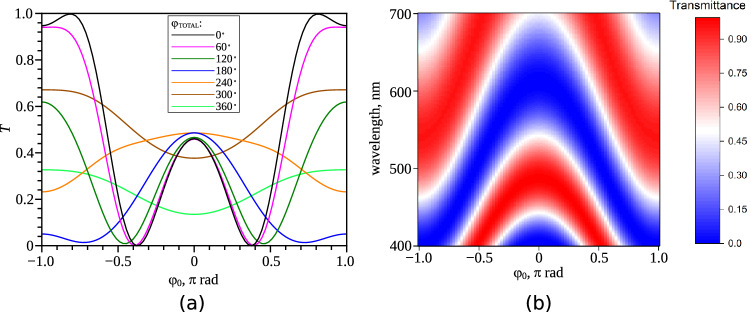
The dependencies of the transmittance *T* of light ($\lambda =546$ nm) passed through the LC cell in the crossed circular polarisers on the angle $\varphi _0$ obtained for the various twist angles of the director $\varphi _{TOTAL}$ (**a**). The dependencies of the transmittance *T* on the angle $\varphi _0$ and light wavelength $\lambda$ obtained for $\varphi _{TOTAL}=60^\circ$ (**b**). The calculations were performed by the Berreman transfer-matrix method for the angles $\alpha = 5.33^\circ$, $\theta _{d/2} = 40^\circ$, LC layer thickness $d=12.7\,\upmu$m, and the refractive indices of LN-396.

Figures 2 and 3 show that at the oblique incidence of light, the interference pattern observed in crossed polarisers will be symmetric with respect to the angle $\varphi _0=0^\circ$ ($\varphi _0=180^\circ$). Figure 4a shows the dependencies of the transmittance *T* of light passed through the LC cell in the crossed circular polarisers on the angle $\varphi _0$ obtained for the various twist angles of the director $\varphi _{TOTAL}$. The calculation was made by the Berreman transfer-matrix method for the angles $\alpha =5.33^\circ$, $\theta _{d/2}=40^\circ$, sample thickness $d=12.7\,\upmu$m, light wavelength $\lambda =546$ nm, and the refractive indices of nematic LN-396. The dependence $T(\varphi _0)$ is symmetric with respect to the angle $\varphi =0^\circ$ (Fig. 4a) regardless of the light wavelength (Fig. 4b). The symmetry $T(\varphi _0)$ is retained even in the case of violation of the Mauguin regime, which for the used sample parameters occurs at $\varphi _{TOTAL}>65^\circ$.

The above mentioned results have been obtained considering the tilt director angle $\theta$ is constant, and the dependence of azimuthal angle $\varphi$ on the coordinate *z* is linear. In general, these conditions are not satisfied. So, for instance, in the twisted nematic structure with the considerable director pre-tilt angles on the substrates, the tilt angle $\theta$ depends on the *z* coordinate. Simultaneously, the dependence of azimuthal director angle on the *z* coordinate deviates from the linear law^48^. Nevertheless, the results obtained for the simple cases show that the maximal value $\delta$ is achieved at $\varphi _0(x,y) = 0^\circ$ regardless of the angles $\theta _{d/2} \le (90^\circ -\alpha )$ and $\varphi _{TOTAL}$. Consequently, that conclusion can be applied for inhomogeneous tilt and nonuniform azimuthal twist angle when the director distribution is symmetric relatively the LC layer centre. The analogous situation is realised in the LC cells under the identical boundary conditions without external forces (e.g. electric field). Further, this method will be employed to analyse the boojums forming in nematic and chiral nematic under conical boundary conditions.

### The director orientation near boojums in LC under conical boundary conditions

#### Achiral nematic liquid crystal

Unless an azimuthal orientation of director is assigned on the substrates (degenerate tangential or conical boundary conditions), the point defects at the surface (boojums) get formed in the system. Near the boojums the azimuthal angle of the director can be represented as $\varphi =m \zeta + \xi$, and orientation **n** at the surface (plane $z = d/2$) can be described by^49^:

7 $$\begin{aligned} \left\{ \begin{array}{l} n_x = \sin {\theta _{d/2}} \cos {(m \zeta + \xi )}\\ n_y = \sin {\theta _{d/2}} \sin {(m \zeta + \xi )}\\ n_z = \cos {\theta _{d/2}} \end{array} \right. , \end{aligned}$$

where $\zeta$ is the angle of **r** in the horizontal plane (see Fig. 1b), *m* is the surface topological charge (the strength) of the defect, $-\pi \le \xi < \pi$ is the phase of the boojum. The azimuthal angle of the director is equal to $\xi$ at $\zeta =0$. The defect strength *m* and the phase $\xi$ are used to classify the boojums (as well as topological defects of other types). Consequently, these two parameters are sufficient to characterise the topological defects^49^. LC cell consists of two substrates and the forming of boojum at one of them (e.g. top one at $z = d/2$) must be accompanied by the formation of the point defect at other (the bottom one at $z = -d/2$). Then an identical azimuthal distribution of director near the boojums at top and bottom substrates (escaped state) corresponds to the minimum of elastic energy. To classify (to determine the strength *m* and phase $\xi$) this pair of boojums they are needed to be considered in the identical coordinate systems, where the *Oz* axis is directed perpendicularly to the LC layer (*xOy* plane) outward. In this case, the boojum with the phase $\xi _{d/2}$ at the top substrate (the subscript is indicated the value of the coordinate *z*) fits to the boojum with the phase $\xi _{-d/2} = (\pi - \xi _{d/2})$ at the bottom substrate (see Supplementary Figs. S1, S2). For example, the boojums with strength $m = \pm 1$ frequently arise in nematics. Then, the boojum with $m = +1$ having the phase $\xi _{d/2} = 0$ at the top substrate (“hyperbolic boojum”) fits to the boojum with $\xi _{-d/2} = -\pi$ (“radial boojum”) at the bottom substrate and vice versa. At that the boojums with $\xi _{d/2} = \pm \pi /2$ at one substrate fit to the identical boojums with $\xi _{-d/2} = \pm \pi /2$ at the second substrate. The analogous situation is observed for the boojums with strength $m = -1$.

**Figure 5:**
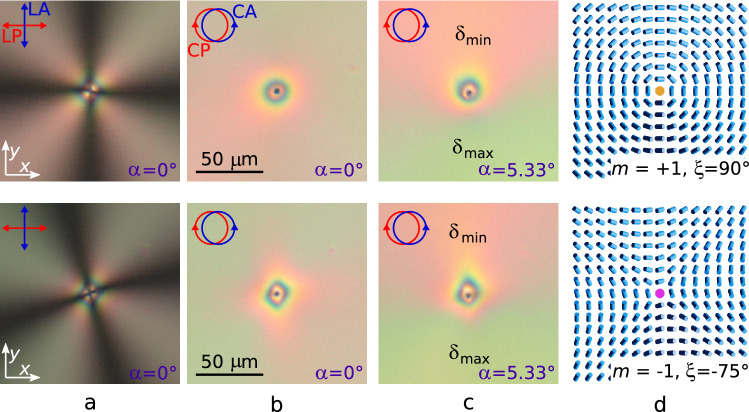
POM photos of nematic with boojums of strength $m = +1$ and the phase $\xi _{d/2} = 90^\circ$ (the first row), $m = -1$ and $\xi _{d/2} = 75^\circ$ (the second row). The photos were taken in the crossed linear polariser (LP) and analyser (LA) at the polariser orientation $0^\circ$ (**a**), in the crossed circular polariser (CP) and analyser (CA) at the angles $\alpha = 0^\circ$ (**b**) and $\alpha = 5.33^\circ$ (**c**). The appropriate director orientations on the top substrate calculated by Eq. (7) (**d**).

To define the strength *m* of defect, the LC cell is examined in the crossed linear polarisers (LP). In this case, extinction bands whose number *N* correlates with the defect strength as $|m|=N/4$ are observed. The strength *m* sign can be determined by means of rotating the polariser relative to the sample^50^. If the rotation directions of the extinction bands and polarisers coincide, then the strength *m* is positive. Vice versa, if the rotation directions are opposite, then *m* is negative. The strength sign of defects with $|m|=1$ can be determined by examining them in the crossed (parallel) circular polarisers (CP). For the boojum with $m = +1$ the interference pattern near the defect observed in the crossed circular polarisers has the radial symmetry, and for the boojum with $m = -1$ the interference pattern has lower order symmetry (Fig. 5b) due to the inequality of splay, band, and twist elastic constants of LC. Since the director azimuthal angle around the boojums varies in the range of $-180^\circ \le \varphi (x,y) = m\zeta +\xi <180^\circ$, then the phase $\xi$ can be found by the oblique light incidence method. Then, for the scheme shown in Fig. 1b,c, $\delta _{max}$ will be achieved at $(m\zeta +\xi ) = 0$, and $\delta _{min}$ satisfies the condition $(m\zeta +\xi ) = -\pi$. In this case, it is convenient to use circular polarisers in the experiment.

The interference pattern observed in the crossed circular polarisers is determined by only the $\delta$ phase difference between ordinary and extraordinary waves. It is seen, that the interference pattern near the boojums satisfy the case when the phase difference rises from zero to $\delta > 4\pi$ (at green light) with increasing distance to the boojum from 0 to approximately 30 $\upmu$m (Fig. 5b). It agrees with the LC cell parameters of $d = 14.6\,\upmu$m thickness and the tilt director angle $\theta _{d/2} \cong 40^\circ$, for which the phase difference is $\delta = 4.17\pi$ (calculated from Eq. (2) at $\lambda =546$ nm) and corresponds to the above mentioned situation when the escaped state is realised in LC bulk. For the boojum with $m = +1$ (Fig. 5 (top row)) the extinction bands form the cross oriented parallel to the crossed linear polarisers. The interference pattern in the circular polarisers is radial symmetry at the normal light incidence ($\alpha = 0^\circ$). The symmetry of the interference pattern decreases when the oblique light incidence ($\alpha = 5.33^\circ$). The interference pattern is symmetric about the line corresponding to the angles $\varphi _0=0^\circ$ ($\delta _{max}$) and $\varphi _0=180^\circ$ ($\delta _{min}$) (Fig. 5c (top row)). For various phase difference in the range from $\delta _{min}$ to $\delta _{max}$, the observed colours of the optical pattern will correspond to the Michel-Lévy interference chart^51^. At normal incidence of light $\Delta n = 0.078$ ($\lambda = 546$ nm) and path difference is $\Gamma = d \Delta n \cong 1140$ nm. Accordingly, the green colour of the interference pattern corresponds to a larger phase difference $\delta _{max}$ ($\Delta n = 0.097$, $\Gamma \cong 1400$ nm), and the pink colour corresponds to $\delta _{min}$ ($\Delta n = 0.060$, $\Gamma \cong 880$ nm). These are consistent with the colour distribution of the interference pattern near the boojum, where value $\Gamma$ increases from 0 at the location of the defect to 1140 nm far from it (Fig. 5b). Colour distribution of the interference pattern allows concluding that $\delta _{max}$ fits to the extinction band oriented at the angle $\zeta = -90^\circ$ and, hence, to the boojums with the $90^\circ$ phase at both the top and bottom substrates.

The twist angle of the boojum with $m = -1$ (Fig. 5 (bottom row)) can be found similarly. In the crossed linear polarisers (at $\alpha = 0^\circ$) the extinction bands form the cross oriented at $15^\circ$ angle to the direction of the linear polariser LP (*Ox* axis). Examining the sample in crossed circular polarisers at oblique light incidence angle $\alpha = 5.33^\circ$ reveals that $\delta _{max}$ fits to the extinction band oriented at $\zeta = -75^\circ$ angle. It corresponds to $\xi _{d/2} = -75^\circ$ phase of the boojum at the top substrate and $\xi _{-d/2} = -105^\circ$ at the bottom one.

The proposed method is applicable for any phase value of boojum, even when the angle $\xi (r)$ depends on the $\mathbf {r}$ distance (see Supplementary Fig. S3). It should be noted that in the studied sample all the boojums with $m = +1$ have the phase close to $\pm 90^\circ$. In other words, these are energetically the most favourable defects for LC LN-396 at the conical anchoring with the $\theta _{d/2} = 40^\circ$ tilt director angle. At that, the various phase values $\xi$ are observed for the boojums with $m = -1$. This is due to the director field distribution near the boojum with $m = -1$ at the various $\xi$ differs by the orientation of symmetry axis only, i.e. the boojum with phase $\xi$ can be obtained by rotating the whole director structure (coordinate system) of the boojum with $\xi _0 = 0^\circ$ by angle $\xi /2$ relative to the *Oz* axis^49^. Thus, the director field’s total free energy near the boojums with $m = -1$ doesn’t depend on the value of $\xi$ phase.

#### Chiral nematic

**Figure 6:**
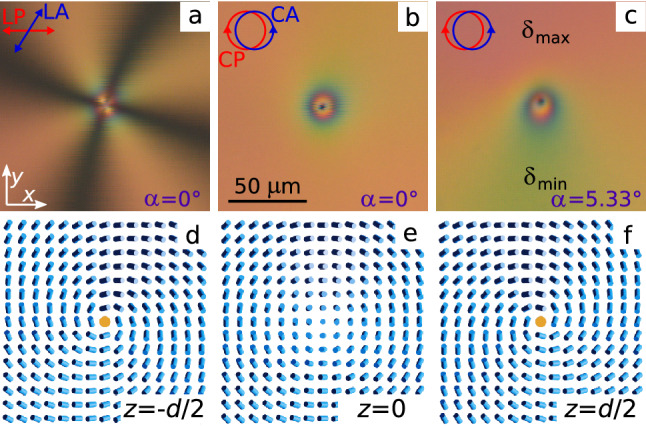
POM photos of chiral nematic with boojum of strength $m = +1$ and the phase on the top substrate $\xi _{d/2} = -106^\circ$ taken in the crossed at angle $58^\circ$ LP and LA at the LP polariser orientation $0^\circ$ (**a**), in the crossed circular polarisers at the light incidence angle $\alpha = 0^\circ$ (**b**) and $\alpha = 5.33^\circ$ (**c**). The corresponding director field on the bottom substrate (**d**), at the layer centre (**e**) and on the top substrate (**f**) calculated by Eqs. (7) and (4).

**Figure 7:**
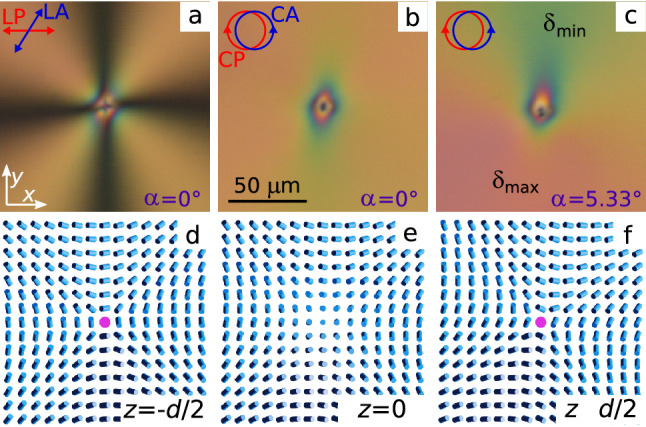
POM photos of chiral nematic with boojum of strength $m = -1$ and the phase on the top substrate $\xi _{d/2} = -116^\circ$ taken in the crossed at angle $58^\circ$ LP and LA at the polariser orientation $0^\circ$ (**a**), in the crossed circular polarisers at the light incidence angle $\alpha = 0^\circ$ (**b**) and $\alpha = 5.33^\circ$ (**c**). The corresponding director field on the bottom substrate (**d**), at the layer centre (**e**) and on the top substrate (**f**) calculated by Eqs. (7) and (4).

The structures of chiral nematic have been studied in the cell of $d = 12.7\,\upmu$m thickness and ratio $d/p = 0.18$. The extinction bands are observed when the angle between the linear analyser and polariser is $58^\circ$, that corresponds to the total twist angle of director $\varphi _{TOTAL} = -32^\circ$ (the left-handed chiral nematic) (Figs. 6a, 7a). Accordingly, the Mauguin number *M* is equal to 14.7 at $\lambda = 700$ nm ($n_{\parallel }=1.720, n_{\bot }=1.520$) for this sample. The boojum with $m = +1$, whose extinction bands form the cross oriented at the angle $-16^\circ$ to the polariser is shown in Fig. 6. The interference pattern observed in the crossed circular polarisers at normal light incidence ($\alpha = 0^\circ$) is an axially symmetric one (Fig. 6b). In this case, the path difference is $\Gamma = 990$ nm. Accordingly, at the oblique light incident, the pink colour of the interference pattern corresponds to a larger phase difference $\delta _{max}$, and the yellow-blue colour corresponds to $\delta _{min}$. The $\delta _{max}$ value (Fig. 6c), and consequently, $\varphi _0(x,y) = 0$ is obtained for the polar angle $\zeta \cong 90^\circ$ at oblique light incidence. From Eq. 4, the phase of boojum at the top substrate is found by $(+1\cdot 90^\circ + \xi _{d/2} = \varphi _{TOTAL}/2)$, hence $\xi _{d/2} = -106^\circ$. The azimuthal director angle at the bottom substrate is smaller by value $\varphi _{TOTAL}$ in comparison with the angle at the top substrate, that corresponds to the phase of boojum $\xi _{-d/2} = -106^\circ = \xi _{d/2}$. This phase value $\xi _{d/2} = \xi _{-d/2} = -90^\circ + \varphi _{TOTAL}/2$ is observed for all boojums with strength $m = + 1$, formed in chiral nematic LN-396 doped with cholesteryl acetate under conical boundary conditions. The boojum phase determined from the experiment at oblique light incidence agrees with the orientation of extinction bands observed in the crossed linear polarisers. Namely, the extinction bands orientation fits to the azimuthal director angle at the bottom substrate $\varphi _{-d/2}$ equal to $0^\circ ,\, \pm 90^\circ$ and $-180^\circ$. It corresponds to the polar angles $\zeta = -106^\circ , -16^\circ , 74^\circ$ and $164^\circ$, respectively when $\xi _{d/2} = -106^\circ$. Fig. 6d,e demonstrates the corresponding director orientations at the bottom substrate ($z = -d/2$), at the layer centre ($z = 0$) and at the top substrate ($z = d/2$). When calculating the director configuration in the layer centre, the approximation for the tilt angle $\theta _0(r) = (10/9)\tan ^{-1}{(r/r_c)}$ at $r \le r_c$ and $\theta _0(r) = \theta _{d/2}$ at $r > r_c$ was used. Where the value $r_c \cong 2d$ was taken in accordance with the experimental data.

The phases of the boojums with $m = -1$ can be determined similarly (Fig. 7). In the crossed linear polarisers the extinction bands form the cross oriented at an angle of approximately $5^\circ$ to the polariser. At oblique light incidence the maximum phase difference $\delta _{max}$ is observed at angle $\zeta \cong -100^\circ$. The phase of boojum at the top substrate is found by the ratio $(-1\cdot (-100^\circ ) + \xi _{d/2} = \varphi _{TOTAL}/2)$, hence $\xi _{d/2} = -116^\circ$. At the bottom substrate, it fits to the boojum with $\xi _{-d/2} = -96^\circ$.

**Figure 8:**
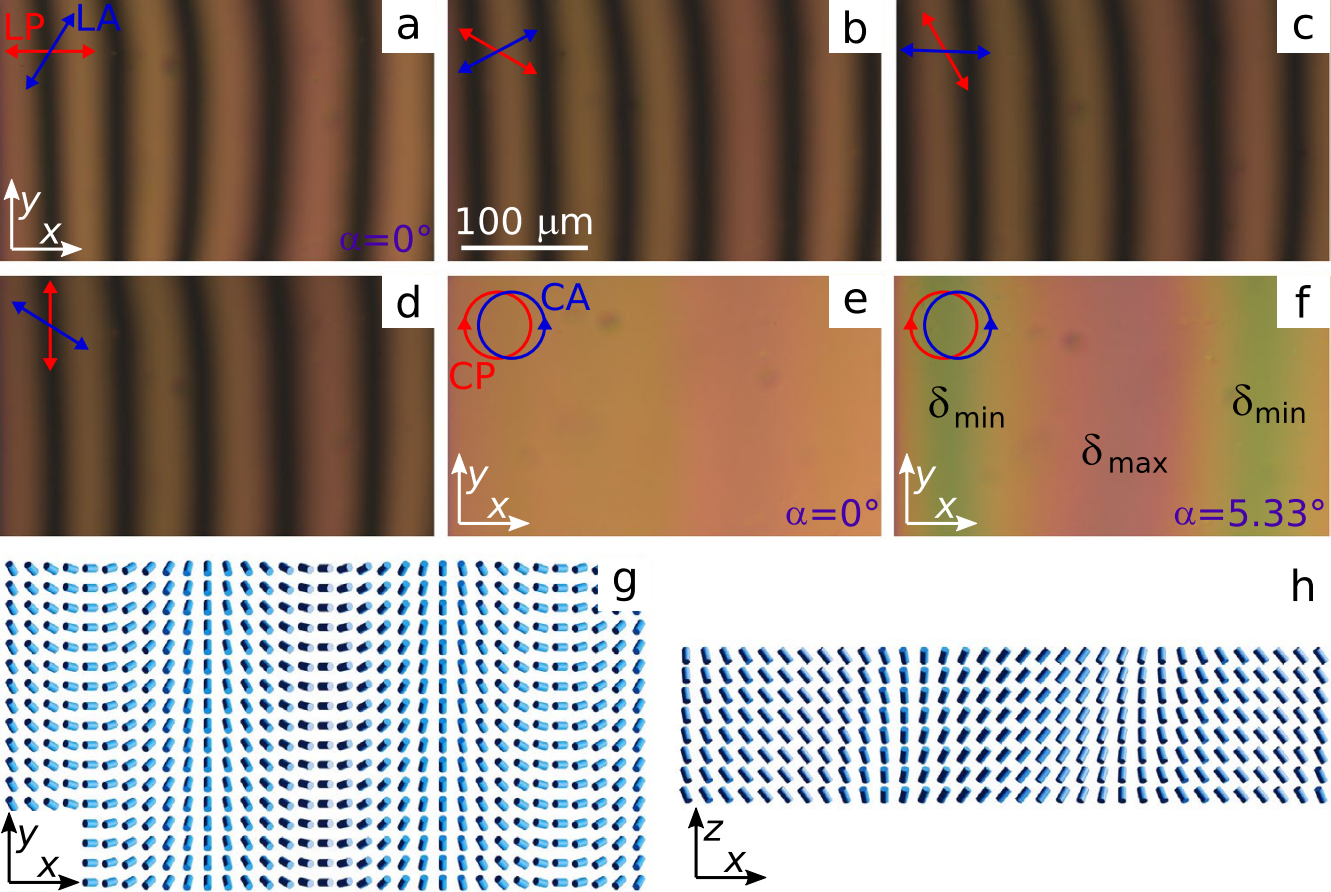
POM photos of the sample area of chiral nematic taken in the crossed at angle $58^\circ$ LP and LA at the polariser orientation $0^\circ$ (**a**), $-30^\circ$ (**b**), $-60^\circ$ (**c**), $-90^\circ$ (**d**), and in the crossed circular polarisers at the light incidence angle $\alpha = 0^\circ$ (**e**) and $\alpha = 5.33^\circ$ (**f**). The corresponding director field at the bottom substrate (**g**) and in the cross-section of LC layer (**h**).

In a chiral nematic under conical boundary conditions, the formation of a defectless structure in which the azimuthal angle of the director changes in the sample plane (xOy plane) is possible. So, Fig. 8 shows the sample area where the azimuthal director orientation doesn’t depend on the *y* coordinate, while it changes along the *Ox* axis almost according to the linear law. When the linear polarisers are crossed at angle $58^\circ$, we observe the set of parallel extinction bands and their position is dependent on the polariser orientation (Fig. 8a–d). At oblique light incidence $\delta _{min}$ ($\varphi _0(x) = -180^\circ$) is reached near the leftmost and rightmost extinction bands in Fig. 8a, and $\delta _{max}$ ($\varphi _0(x) = 0$) meets to the area close to the central extinction band. Figure 8g,h demonstrates the corresponding director distributions at the bottom substrate and in the cross-section of LC layer.

## Discussion

Thus, we have studied the director configuration and the surface point defects formed in the chiral and achiral nematic under conical anchoring. The oblique light incidence method has been developed to determine the director orientation by the polarising microscopy techniques. At the Mauguin’s waveguide regime and oblique light incidence the $\delta _{max}$ phase difference between ordinary and extraordinary waves is reached for the director azimuthal angle $\varphi _0(x,y) = 0$ independently of the $\varphi _{TOTAL}$ total twist angle of chiral nematic. The boojums formed in chiral and achiral nematic with the $\theta _{d/2} \cong 40^\circ$ tilt angle of director at the interface have been examined. It has been found, that the boojums with $m = +1$ having the phase $\xi = \pm 90^\circ$ and $\xi = (-90^\circ + \varphi _{TOTAL}/2)$ are formed respectively in achiral and chiral LC mixtures based on LN-396 nematic. At that, the forming boojums with strength $m = -1$ can have different phase values. The defectless structure with the periodically varied azimuthal director angles on the substrates has been studied too. The obtained results are of interest to researchers of the periodic or/and the defect structures, and the proposed oblique light incidence method can be applied to analyse the structures formed in other uniaxially oriented systems with the tilted or hybrid surface anchoring.

## Materials and methods

The nematic mixture LN-396 (Belarusian State Technological University) and LN-396 doped with the left-handed chiral additive cholesteryl acetate (Sigma Aldrich) were used as a nematic and chiral nematic, respectively. The concentration of cholesteryl acetate is 0.2%, it satisfies to the helix pitch of cholesteric $p = 72.5\,\upmu$m^39^. The experiment was carried out with sandwich-like cells consisting of two glass substrates coated with the poly(isobutyl methacrylate) (PiBMA) (Sigma Aldrich) films. The polymer films were deposited on the substrates by spin coating without further treatment. In our previous works were found that for the nematic LN-396 the PiBMA specifies the conical boundary conditions with the tilt angle $\theta _{d/2} \cong 40^\circ$ and azimuthal degeneration^39,52^. The LC layer thickness *d* assigned by the glass microspheres was measured by means of the interference method before the filling process. LC cells were filled at the room temperature and were kept for at least 24 h before measurements. The study was carried out using the polarising optical microscope (POM) Axio Imager.A1m (Carl Zeiss) with the long-distance objective 20x/0.22. The refractive indices of LC LN-396 are $n_{\perp } = 1.528, n_{\parallel } = 1.741$ ($\lambda = 546$ nm). The transmittance of the structure at oblique light incidence for crossed circular polarisers were calculated by the Berreman method–the transfer-matrix method generalized for an anisotropic medium^53,54^. The transmittance dependencies on the $\varphi _0$ angle were calculated by rotating the liquid crystal layer by an angle of $2\pi$ about the *z*-axis. In calculations, the wave was incident at the LC layer from glass with a refractive index of $n_{glass}=1.5$.

## Supplementary Information

Supplementary Figures.

## Data Availability

All data generated and analyzed during this study are available upon request.
