# Supplementary material for: Oblique light incidence method to study topological defects in nematic layers with conical boundary conditions
Source: Sci Rep. 2021 Aug 31;11:17433. doi: 10.1038/s41598-021-96784-9 (PMC8408232; doi:10.1038/s41598-021-96784-9)

Supplementary Information

Oblique light incidence method to study topological defects in nematic layers with conical boundary conditions

Mikhail N. Krakhalev*[1,2]

[1] *Kirensky Institute of Physics, Federal Research Center KSC SB RAS, Krasnoyarsk 660036, Russia*

[2] *Institute of Engineering Physics and Radio Electronics, Siberian Federal University, Krasnoyarsk 660041, Russia*

Corresponding author: M.N. Krakhalev, e-mail: kmn@iph.krasn.ru

Supplementary Figure 1-3

Supplementary Figure 1| Schemes of the director field around a pair of $m = +1$ boojums corresponding to the escaped state and located on the top (top view) and bottom (bottom view) substrates. The $\xi_{d/2}$ phase of boojum on the top substrate is 0 (a), π/4 (b), π/2 (c), 3π/4 (d), -π (e), -3π/4 (f), -π/2 (g), -π/4 (h). The tilt angle at substrates is $\theta_{d/2}$ = 40º.

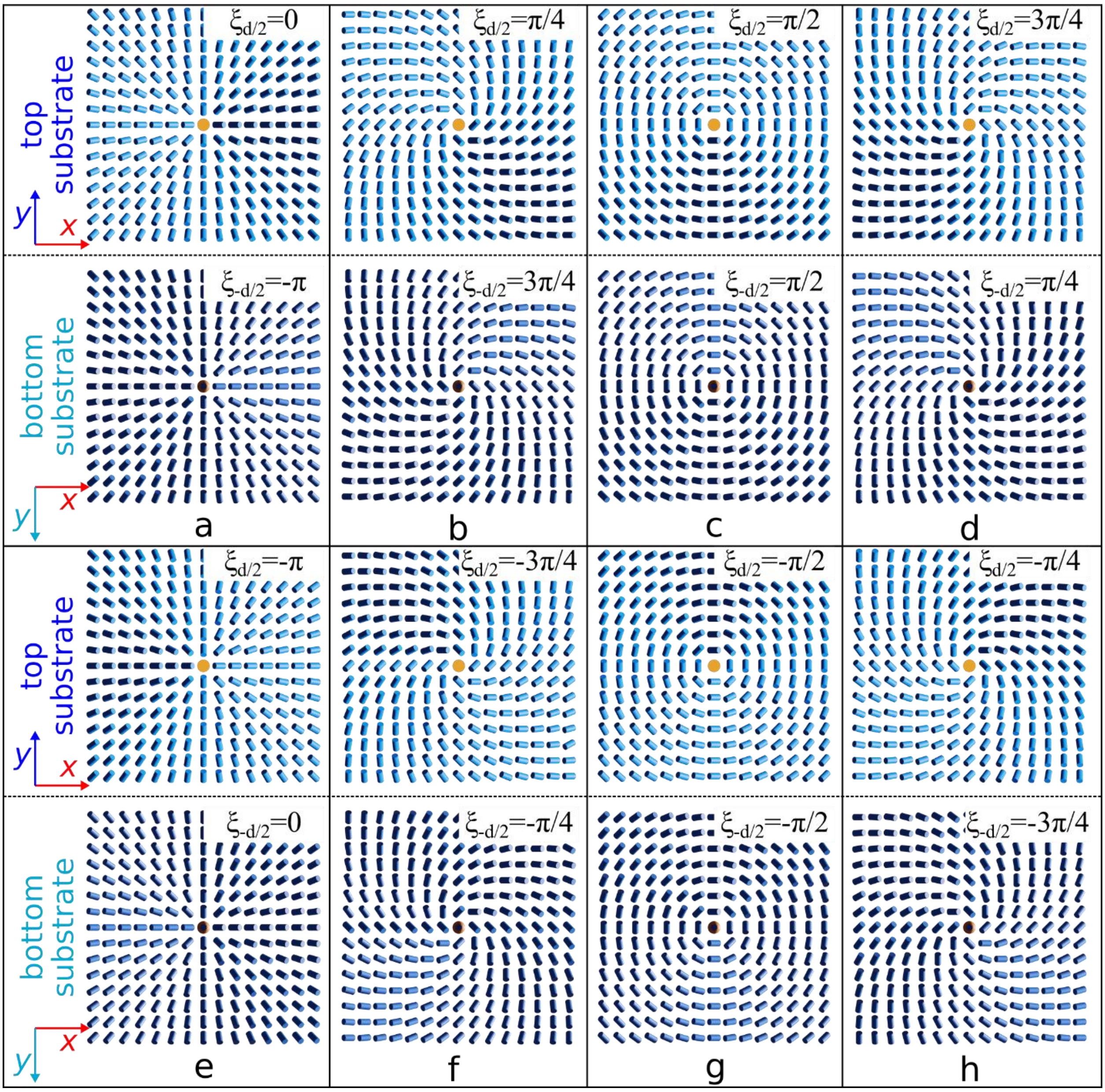

Supplementary Figure 2| Schemes of the director field around a pair of $m = -1$ boojums corresponding to the escaped state and located on the top (top view) and bottom (bottom view) substrates. The $\xi_{d/2}$ phase of boojum on the top substrate is 0 (a), $\pi/4$ (b), $\pi/2$ (c), $3\pi/4$ (d), $-\pi$ (e), $-3\pi/4$ (f), $-\pi/2$ (g), $-\pi/4$ (h). The tilt angle at substrates is $\theta_{d/2} = 40°$.

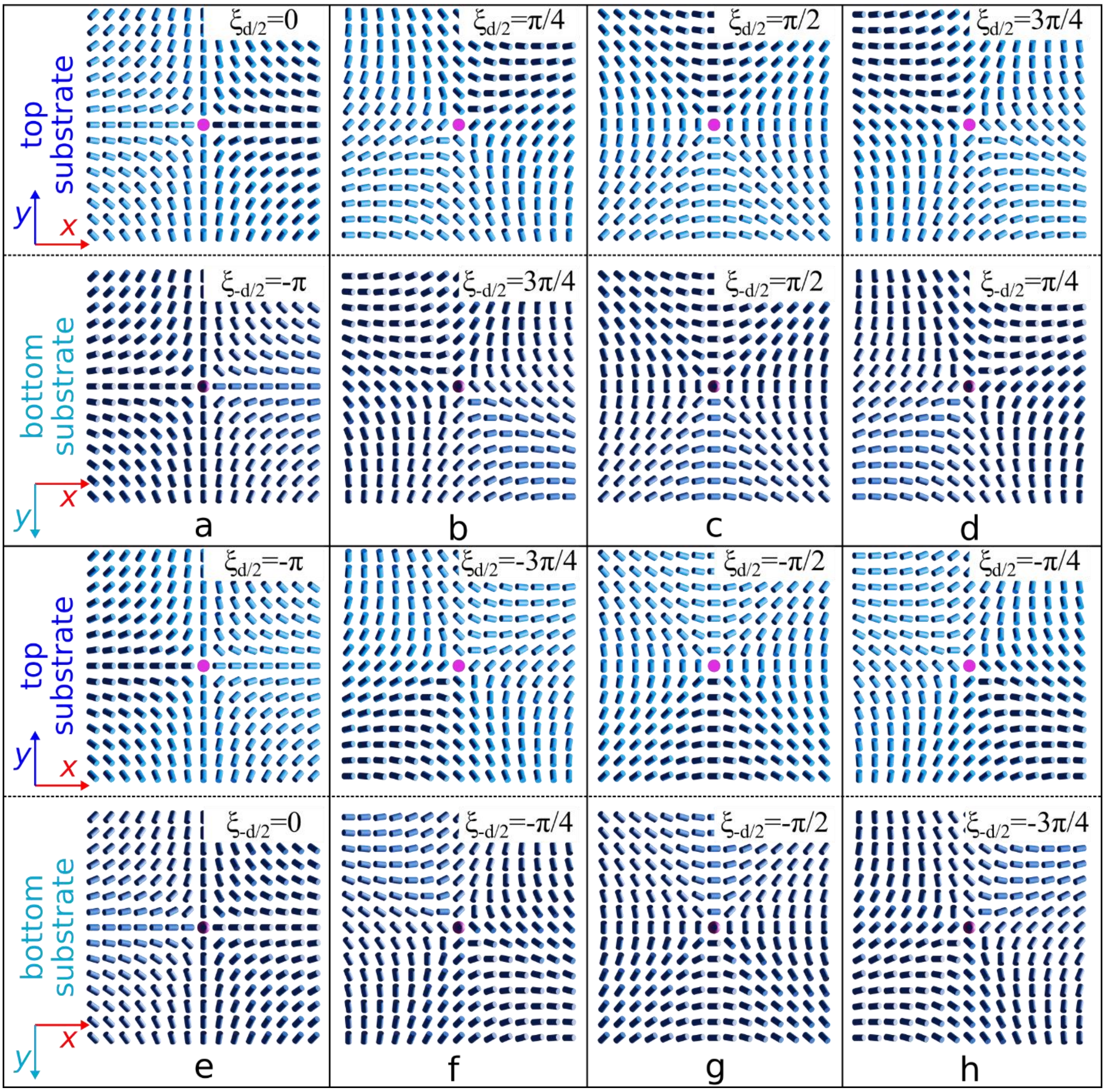

Supplementary Figure 3| POM photos of nematic with boojums of $m = +1$ strength and the $\xi_{d/2} = 105^{o}$ phase (a), $m = +1$ and $\xi_{d/2} = -110^{o}$ (b), $m = -1$ strength and the $\xi_{d/2} = 55^{o}$ phase (c), and $m = -1$ and $\xi_{d/2} = -70^{o}$ (d). The photos were taken in the crossed linear polariser (LP) and analyser (LA) at the polariser orientation 0º (top row), in the crossed circular polariser (CP) and analyser (CA) at the angle $\alpha = 5.33^{o}$ (bottom row).

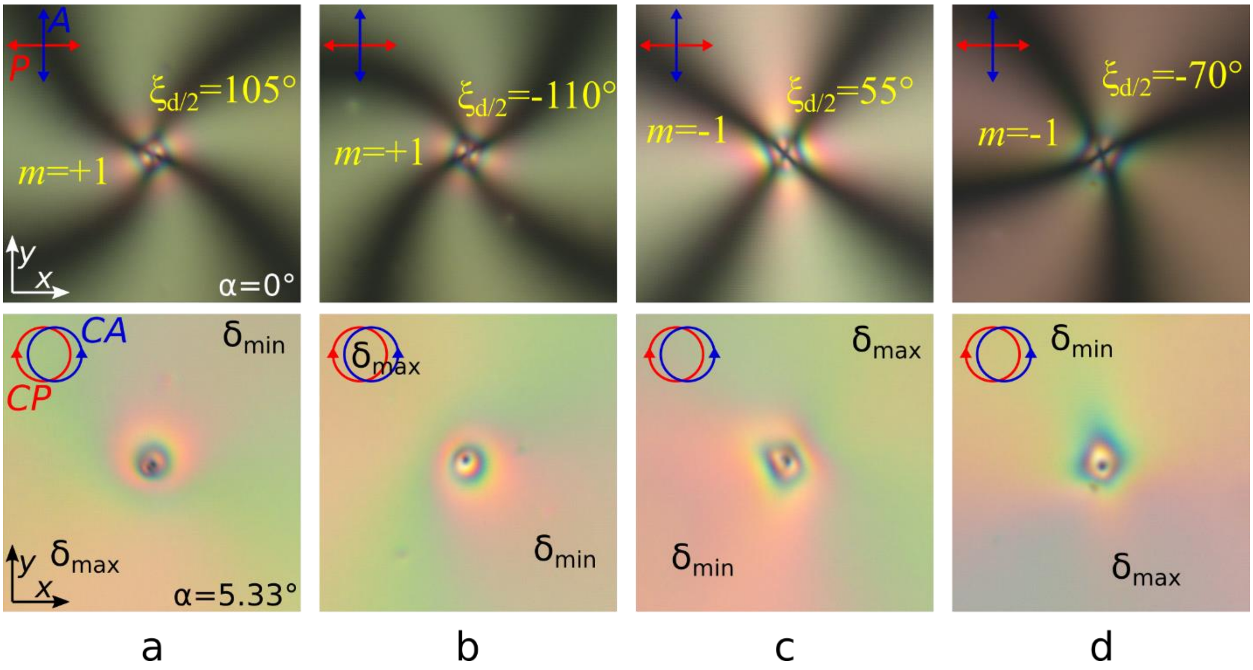

Supplement: Supplementary file 1 — Supplementary Figures. [file 41598_2021_96784_MOESM1_ESM.pdf]
